# Enhancing anomaly detection in plant disease recognition with knowledge ensemble

**DOI:** 10.3389/fpls.2025.1623907

**Published:** 2025-08-15

**Authors:** Jiuqing Dong, Heng Zhou, Alvaro Fuentes, Sook Yoon, Dong Sun Park

**Affiliations:** ^1^ School of Computer and Information Engineering, Institute for Artificial Intelligence, Shanghai Polytechnic University, Shanghai, China; ^2^ Department of Electronic Engineering, Jeonbuk National University, Jeonju, Jeollabuk-do, Republic of Korea; ^3^ Core Research Institute of Intelligent Robots, Jeonbuk National University, Jeonju, Jeollabuk-do, Republic of Korea; ^4^ Department of Computer Engineering, Mokpo National University, Muan-gun, Jeollanam-do, Republic of Korea

**Keywords:** anomaly detection, plant disease recognition, few-shot learning, knowledge fusion, transfer learning

## Abstract

Plant diseases pose a significant threat to agriculture, impacting food security and public health. Most existing plant disease recognition methods operate within closed-set settings, where disease categories are fixed during training, making them ineffective against novel diseases. This study extends plant disease recognition to an open-set scenario, enabling the identification of both known and unknown classes for real-world applicability. We first benchmark the anomaly detection performance of three major visual frameworks—convolutional neural networks (CNNs), vision transformers (ViTs), and vision-language models (VLMs)—under varying fine-tuning strategies. To address the limitations of individual models, we propose a knowledge-ensemble-based method that integrates the general knowledge from pre-trained models with domain-specific knowledge from fine-tuned models in the logit and feature spaces. Our method significantly improves over existing baselines. For example, on vision-language models with 16-shot per class, our approach reduces the FPR@TPR95 from 43.88% to 7.05%; in the all-shot setting, it reduces the FPR@TPR95 from 15.38% to 0.71%. Extensive experiments confirm the robustness and generalizability of our approach across diverse model architectures and training paradigms. We will release the code soon at https://github.com/JiuqingDong/Enhancing_Anomaly_Detection.

## 1 Introduction

Protecting crops from plant diseases plays a significant role in meeting the growing demands for food quality and quantity. Direct yield losses caused by pathogens, pests, and weeds result in a total loss of 20% to 40% of global agricultural productivity Oerke (2006). In reality, this value does not fully reflect the true cost to consumers, public health, and farmers caused by crop losses. This is because plant diseases often come with a decline in crop quality, including nutrients, taste, and appearance defects, among other additional costs Savary et al. (2012). To mitigate these impacts, early detection of plant diseases and taking remedial measures are effective in current crop protection Sarkar et al. (2023); Xu et al. (2024); Dong et al. (2022).

**Figure 1:**
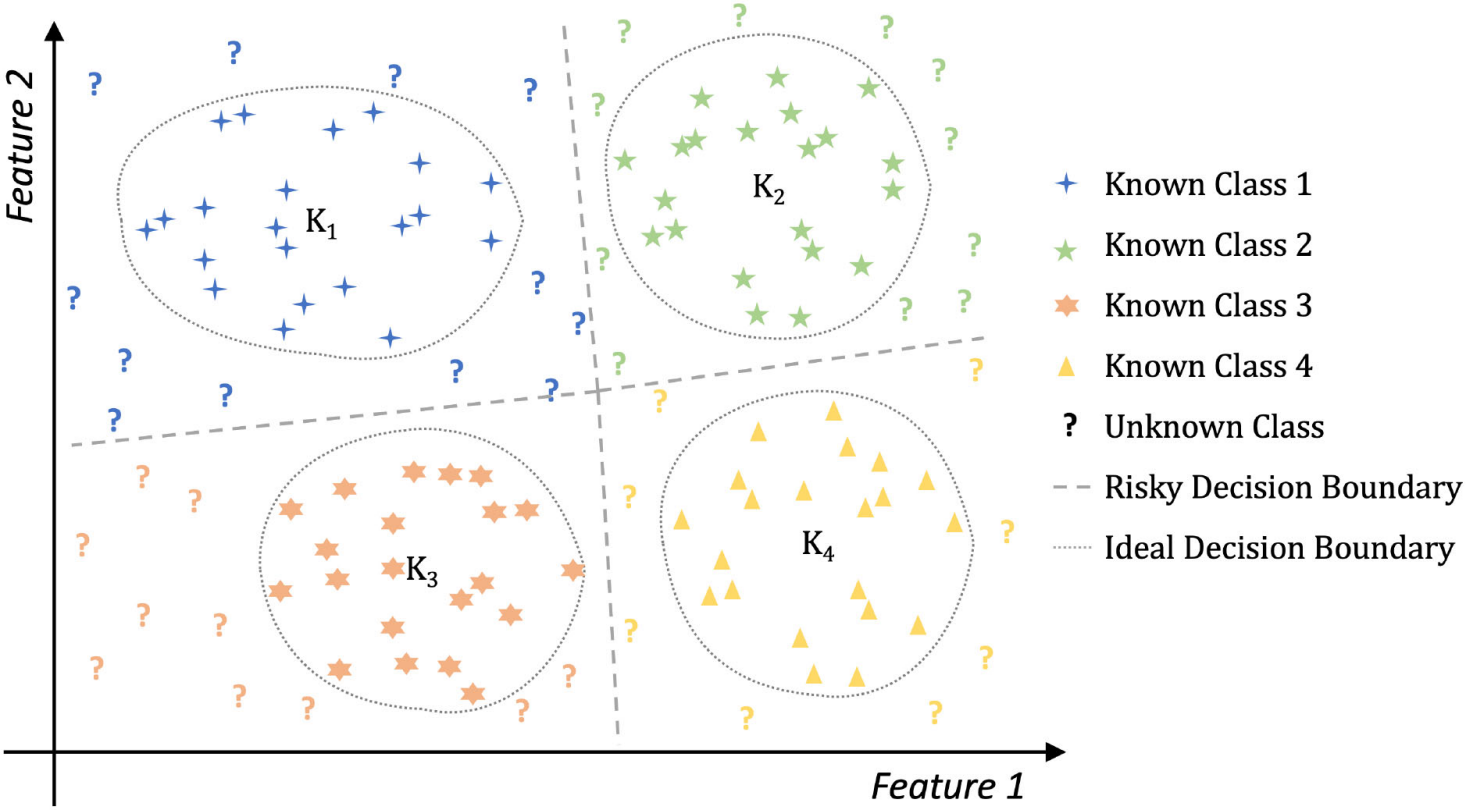
The decision risk in closed-set learning. Algorithms based on closed-set learning will produce a risky decision boundary that may misclassify certain unknown instances as belonging to known classes. Feature 1 and Feature 2 are abstract axes representing two dimensions in the feature space. They do not correspond to specific semantic features.

By leveraging advanced deep neural network frameworks Chai et al. (2021) and transfer learning techniques Iman et al. (2023), plant disease recognition performance has significantly improved. However, existing methods assume that the categories in the test datasets are entirely consistent with those in the training sets, which is unrealistic in real-world plant disease recognition scenarios. Given the variability and complexity of plant diseases, it is impractical to collect samples of all possible disease classes beforehand to train a neural network Meng et al. (2023). In this paper, we refer to classes not included in the training set as unknown or anomalous classes. Existing methods often treat these unknown instances as belonging to known categories, which increases the risk of incorrect decisions in the system. This decision risk is illustrated in **Figure 1**. For example, in the figure, blue question marks are mistakenly classified under *K*_1_, and yellow question marks under *K*_4_. An ideal boundary should exclude these unknown instances while correctly classifying those within known categories. Such a decision mechanism can effectively alert humans to potentially risky examples within the system. Therefore, it is essential to develop plant disease recognition systems capable of identifying unknown disease types, ensuring robust and reliable performance in real-world applications Dong et al. (2023, 2024b, 2025).

Recognizing and rejecting unknown samples is commonly referred to as anomaly detection Cui and Wang (2022); Dong et al. (2023), out-of-distribution(OOD) detection Cui and Wang (2022); Dong et al. (2024c), or open set recognition(OSR) Mahdavi and Carvalho (2021). The term ‘anomaly detection’ is more widely used in applied fields, while ‘out-of-distribution detection’ is typically adopted in theoretical studies. Hence, we uniformly use ‘anomaly detection’ to align with our theme. In this paper, classes present in the training dataset are referred to as ‘known classes’, while those that appear in the test dataset but are absent from the training dataset are referred to as ‘unknown classes’. Both known and unknown classes are included in the test dataset, and our objective is to enable the model to identify and reject these unknown samples.

Recent advances have explored a variety of anomaly detection techniques specifically for agriculture, ranging from CNN-based classifiers on field-acquired images to multimodal integration frameworks. For instance, Mendoza-Bernal et al. proposed a CNN-based pipeline for anomaly detection in weed and crop images under both field and aerial settings, achieving high accuracy across multiple datasets while tackling common issues such as class imbalance and limited generalizability Mendoza-Bernal et al. (2024). Similarly, generative and contrastive approaches, such as f-AnoGAN Schlegl et al. (2019) and AACLIP Ma et al. (2025), have demonstrated strong performance by modeling anomalies beyond closed-set assumptions. Notably, Leygonie et al. explicitly addressed open-set challenges in plant disease detection, highlighting the critical need for systems that can recognize unknown diseases without prior exposure to their categories Leygonie et al. (2024). However, many of these methods rely on architectural modifications, additional modules, or reconstruction-based learning objectives, which are often task-specific and require retraining the entire model from scratch.

Additionally, *post-hoc* anomaly detection methods have gained popularity due to their advantage of not requiring modifications to existing training objectives or pipelines. However, their effectiveness in the specific context of plant disease anomaly detection remains underexplored. To address this gap, our study proposes a readily deployable knowledge integration method aimed at enhancing the anomaly detection capabilities of existing models in agricultural settings. Specifically, we begin by investigating how different training architectures affect the performance of state-of-the-art *post-hoc* anomaly detection algorithms, and we establish a comprehensive benchmark using the PlantVillage dataset Hughes and Salathé (2015). To this end, we apply advanced *post-hoc* detection techniques across three distinct training paradigms: convolutional neural networks (CNNs)Liu et al. (2022), vision transformers (ViTs)Dosovitskiy et al. (2020), and vision-language models (VLMs) Radford et al. (2021). The first two have become standard in recent visual recognition tasks, while the third represents a novel multimodal framework that has gained attention in the past two years.

Fine-tuning strategies also play a critical role in anomaly detection performance. Therefore, we further investigate the impact of different fine-tuning paradigms on anomaly detection. Specifically, for the convolutional neural network and vision transformer models, we implement full fine-tuning, visual adapter tuning Liu and Rajati (2020), and visual prompt fine-tuning Jia et al. (2022). For vision-language models, we examine contextual prompt fine-tuning Zhou et al. (2022b, 2022a), visual prompt fine-tuning Bahng et al. (2022), and dual-modality fine-tuning Zang et al. (2022). Additionally, we explore the performance differences of these anomaly detection methods under limited sample scenarios, referred to as few-shot anomaly detection. Our findings reveal that the performance of the same anomaly detection method varies significantly across different training architectures, fine-tuning paradigms, and sample configurations. Moreover, current state-of-the-art anomaly detection methods based on vision-language models perform poorly on plant disease detection tasks. We attribute this to the weak contextual representation of plant diseases in the text branch of these models.

To address these challenges, we propose a knowledge ensemble method that enhances the anomaly detection performance of baseline methods. Our approach reduces performance discrepancies across different training architectures and fine-tuning paradigms, providing a more robust solution for plant disease anomaly detection. Overall, our main contributions are as follows:

- We establish an anomaly detection benchmark on the Plant Village dataset, and evaluate the performance of classical *post-hoc* detection methods across different training architectures, fine-tuning paradigms, and sample scales. To the best of our knowledge, this is the most comprehensive benchmark for anomaly detection in plant diseases to date.
- We propose a knowledge integration method that combines the general knowledge of pre-trained models with the domain-specific knowledge of fine-tuned models in the logit space. This method significantly improves baseline methods and reduces performance discrepancies across different fine-tuning paradigms and training framework configurations.
- Our method demonstrates outstanding performance across various training architectures, fine-tuning paradigms, and sample scales. Even under limited-sample settings, our method achieves results comparable to those obtained when training on the full dataset.
- Using the vision transformer architecture as an example, we validate the effectiveness of the proposed enhancement method under different data partitioning strategies on additional plant disease recognition datasets.

## 2 Preliminary

### 2.1 Problem statement

In this section, we define the anomaly detection problem. The training set is represented as shown in **Equation 1**:


(1) $$D^{\text{train}}={\left\{\left(x_{i},y_{i}\right)\right\}}_{i=1}^{N},\;i\in \mathbb{N},$$

where *x*, *y*, and *N* denote the input sample, its label, and the total number of images, respectively. The set of known classes is denoted by *K* = {*c*_1_ *, c*_2_ *, c*_3_ *,…,c_k_*}, where *y_i_* ∈ *K*. In few-shot settings, the training set is defined in **Equation 2**:


(2) $$D^{\text{train}}={\left\{\left(x_{i},y_{i}\right)\right\}}_{i=1}^{M\cdot k},$$

where *M* is the number of samples per known class and *k* is the number of known classes. Few-shot scenarios typically involve *M* ∈ {2, 4, 8, 16}.

We further assume the existence of a set of unknown classes, as shown in **Equation 3**:


(3) $$U=\left\{c_{t+1},c_{t+2},\ldots ,c_{t+u}\right\},$$

which do not appear during training but may emerge during inference. To ensure open-set conditions, the known and unknown classes must be disjoint, as required in **Equation 4**:


(4) $$K\cap^{}U=\emptyset .$$

Under this formulation, anomaly detection is modeled as a binary classification task.


(5) $$Decision_{\lambda }\left(x_{i}\right)=\{\begin{array}{ll} Unknown\;Class & S\left(x_{i}\right)>\lambda \\ Known\;Class & S\left(x_{i}\right)\leq \lambda \end{array},$$

where *S*(*x_i_*) represents the uncertainty score for a sample *x_i_*. *A* higher score indicates greater uncertainty, and samples with *S*(*x_i_*) *> λ* are classified as belonging to unknown classes.

### 2.2 Classic *post-hoc* anomaly detection

During testing, we utilize various scoring functions to classify images as either known or unknown. For CNN and ViT frameworks, we apply the maximum logits Basart et al. (2022) and energy-based Liu et al. (2020) scoring methods. For vision-language pre-trained models, we use the MCM score Ming et al. (2022).

Max Logits Basart et al. (2022). The max logits score utilizes the logits of the classification head. The uncertainty score calculated by max logits (*S_ML_*) can be formalized as **Equation 6**:


(6) $$S_{ML}=-\text{max }\left(\frac{z_{i}/T}{\sum_{i=1}^{K}z_{i}/T}\right),$$

where *z_i_* denotes the logits for class *i*, *K* is the number of known classes, and *T* is the temperature scaling factor.

Energy Liu et al. (2020). Similar to the max logit method, the energy-based approach is less susceptible to the issue of overconfidence, thus enabling a more flexible utilization of the classifier to assess the uncertainty of samples. The uncertainty score calculated by energy (*S_energy_*) can be formalized as follows:


(7) $$S_{energy}=-log\left(\sum_{i=1}^{K}e^{z_{i}}/T\right),$$

where *T* = 1 is used as the default temperature scaling factor.

MCM score Ming et al. (2022); Ming and Li (2024). The MCM score utilizes the softmax score of global image features *f* and text features *g*. The uncertainty score calculated by MCM Ming et al. (2022) (*S_MCM_*) can be formalized as follows:


(8) $$S_{MCM}=-\text{max }\left(\frac{e^{\left(\text{similarity}\left(f,g_{i}\right)/T\right)}}{\sum_{i=1}^{K}e^{\left(\text{similarity}\left(f,g_{i}\right)/T\right)}}\right),$$

where *g_i_* denotes the text feature of known class *i*. *K* denotes the number of known classes. The rationale is that, for known samples, the image features *f* of known classes should be closer to the text features *g_i_*, and vice versa.

### 2.3 Evaluation metrics

*FPR@TPR95* Du et al. (2022): This metric computes the false positive rate when the true positive rate reaches 95%. It is defined based on **Equation 9**:


(9) $$TPR=\frac{TP}{TP+FN},\;FPR=\frac{FP}{FP+TN},$$

where TP, FN, FP, and TN represent true positives, false negatives, false positives, and true negatives, respectively.

*AUROC* Muschelli (2020): This metric treats known data as positive and unknown data as negative, generating various TPRs and FPRs at different thresholds to calculate the area under the receiver operating characteristic curve. This metric in anomaly detection tasks indicates the probability that the uncertainty score for unknown data is higher than that for known data. In our formulation, any unknown sample predicted as a known class is considered a misclassification, and vice versa.

*Accuracy:* We assess the accuracy of the known classes, following the method described by Krizhevsky et al. (2012).

## 3 Methodology

In this section, we first establish a benchmark on the Plant Village dataset, covering three training frameworks and classical anomaly detection methods. Furthermore, we propose a novel enhancement method that improves the performance of the original anomaly detection methods from the perspective of feature similarity.

### 3.1 Benchmark of plant disease anomaly detection

Our motivation for constructing a benchmark for plant disease anomaly detection is based on the following points: First, current anomaly detection methods are primarily developed by training on the entire dataset. However, obtaining large amounts of labeled data is sometimes impractical. Secondly, these methods are often not validated across all frameworks during development; some use CNN frameworks for training, while others employ ViT or VLM. Third, in the context of plant disease anomaly detection, there has been no research analyzing the impact of different training frameworks on anomaly detection performance. To fill this gap, we first need to establish a comprehensive benchmark. Our benchmark incorporates different training frameworks, fine-tuning paradigms, sample scales, and anomaly detection methods.

#### 3.1.1 CNN and ViT frameworks

**Figure 2:**
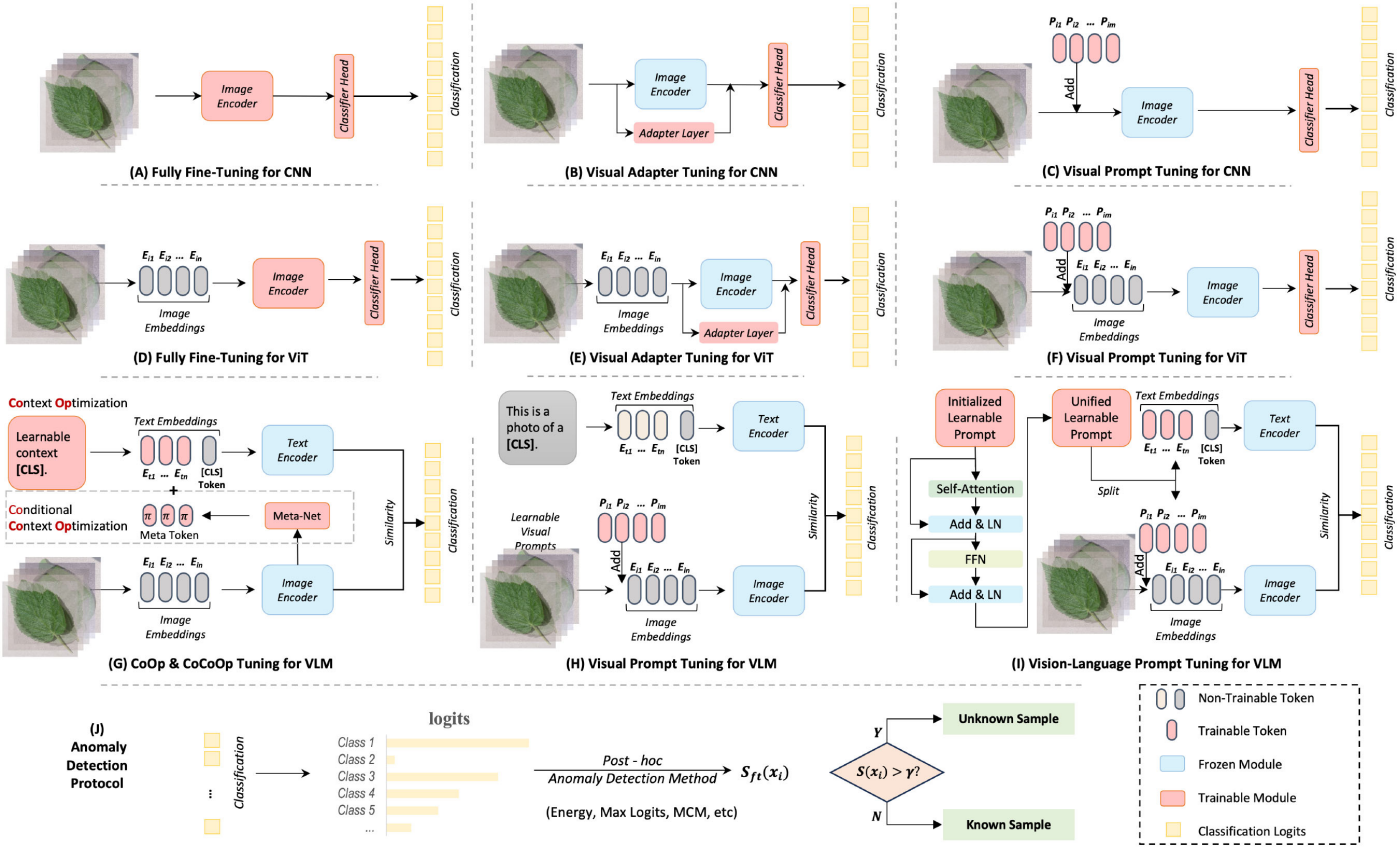
Overview of different training frameworks with fine-tuning paradigms. Subfigures **(A–H)** illustrate sketches of different fine-tuning techniques, while Subfigure **(I)** depicts the process of *post-hoc* anomaly detection methods.

In deep learning, it has been probed that using a pre-trained model can broadly improve performance in downstream tasks, due to its strong generalization capabilities. We first investigate using single-modal visual pre-trained models, such as CNN and ViT, for anomaly detection in plant diseases. Specifically, we explore fully fine-tuning (FFT), visual adapter tuning (VAT), and visual prompt tuning (VPT) in visual models, which are typical fine-tuning methods. The schematic diagrams of these fine-tuning paradigms are shown in **Figures 2A–F**. A brief review of these three techniques is as follows:

Fully fine-tuning refers to the process where all the parameters of a pre-trained neural network are updated during the training phase. This approach assumes that while the pre-trained model has learned general knowledge from its initial training, further adjustments to all of the weights can help the model better adapt to the nuances of the new data or task. However, fully fine-tuning can be computationally expensive and prone to overfitting, especially if the new dataset is small.

Visual adapter tuning is a more parameter-efficient fine-tuning (PEFT) method where instead of updating all the parameters, small adapter modules are inserted into the pre-trained model. Only the parameters of these adapters are updated, while the original network parameters remain frozen, which helps the model to learn task-specific features without overwriting the general knowledge learned during pre-training. This approach is less resource-intensive compared to fully fine-tuning and reduces the risk of overfitting. This study uses a basic multilayer perceptron (MLP) module with a residual connection in Transformer layers, as suggested by Houlsby et al. (2019); Pfeiffer et al. (2020).

Visual prompt tuning is a novel PEFT approach inspired by advances in natural language processing. For visual tasks, prompt tuning involves modifying either the input data or the model architecture by adding a set of trainable parameters (the prompts) while keeping the rest of the model fixed. The idea is to “prompt” the model to apply its pre-trained knowledge in a way that’s useful for the new task. This method is useful for adapting large models to new tasks without the need for extensive re-training. This study follows Jia et al. (2022) approach, inserting ten learnable prompt tokens in each Transformer layer.

#### 3.1.2 Vision-language model

Vision-language models (VLM) are replacing traditional supervised pre-training models [e.g., based on ImageNet Deng et al. (2009)] as the new generation of foundational models for vision tasks. For instance, in early 2021, OpenAI released CLIP Radford et al. (2021), a large-scale multi-modal model designed for aligning images and text. It was pre-trained on 400 million internet image-text pairs, acquiring rich visual-linguistic knowledge through contrastive learning. By using text features as classification weights during the inference phase, CLIP enables zero-shot predictions and can be applied to a variety of downstream tasks in a zero-shot manner. However, CLIP primarily excels in general domain datasets like ImageNet and often underperforms when processing data from certain fine-grained domains Dong et al. (2024a). Therefore, it is necessary to fine-tune the model using specific data from downstream tasks.

Fine-tuning based on textual prompts is a classic approach to addressing the issue of small sample generalization in large language models. Thus, consistent with Ming and Li (2024), we first investigated two representative works, CoOp Zhou et al. (2022b) and CoCoOp Zhou et al. (2022a). CoOp is the first to incorporate the idea of prompt learning into the adaptation of downstream tasks for multimodal pre-trained foundational models. It uses learnable word embeddings to automatically construct contextual prompts instead of manually designing prompt templates for each task. Subsequently, Zhou et al. (2022a) introduced visual features to guide the context optimization, proposing Conditional Contextual Optimization (CoCoOp). CoCoOp constructs a meta-network to learn features from images. These features are then combined with prompt vectors to enhance CoOp’s generalization performance for new category data. Both CoOp Zhou et al. (2022b) and CoCoOp Zhou et al. (2022a) methods only fine-tune the textual side of CLIP. However, a multi-modal model where both visual and textual aspects are equally important. By incorporating visual information as a condition for text optimization, CoCoOp achieved significant improvements. Hence, we argue that visual information may be more effective for plant disease anomaly recognition than textual information. We further investigated the visual side fine-tuning method of CLIP. For visual prompt tuning Jia et al. (2022), we use ‘This is a photo of a [CLS]’ as the textual prompt. For vision-language prompt tuning Zang et al. (2022), we use unified prompt tokens to adapt downstream tasks. Overall, we re-implemented these fine-tuning paradigms for plant disease anomaly detection, including CoOp Zhou et al. (2022b), CoCoOp Zhou et al. (2022a), VPT-CLIP Bahng et al. (2022), and VLPT Zang et al. (2022). The schematic diagrams of these fine-tuning paradigms are shown in **Figures 2G–I**. To ensure a fair comparison, we set the same number of learnable text prompt tokens, with a length of 16.

### 3.2 Uncertainty score based on knowledge ensemble

**Figure 3:**
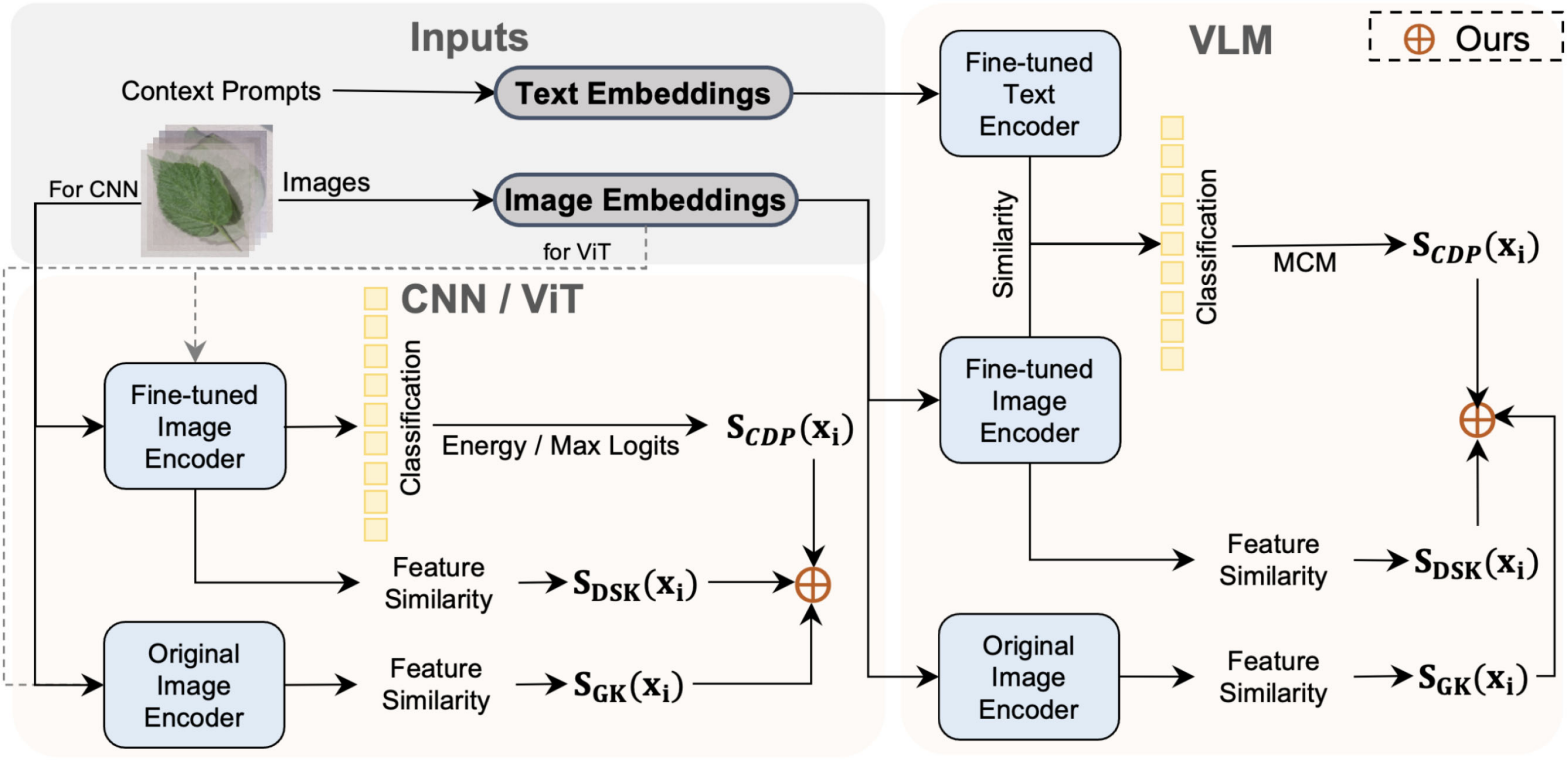
Overview of our ensemble method for anomaly detection in plant disease recognition. The method is applicable to three types of models: CNNs, ViTs, and VLMs. We leverage domain-specific scores *S_CPD_* from fine-tuned models and generalizable scores *S_GK_* from original (frozen) image encoders to perform score-level integration. This strategy enables our method to combine discriminative power with generalization, improving robustness in open-set and few-shot scenarios.

Fine-tuning models on more training samples can acquire more domain-specific knowledge, but inevitably, the model will lose some of the more general knowledge from the original pre-trained model Wortsman et al. (2022). In other words, fine-tuning pre-trained models using specific domain data often comes at the cost of model robustness and generalization in exchange for significant performance improvements on the target domain distribution. Although strategies like freezing the backbone, as in the PEFT method, are effective in preserving general knowledge, the learnable adapters and prompts trained from scratch, cannot encompass all general knowledge in the output features. Therefore, we propose an uncertainty score-based ensemble method. It integrates the uncertainty distribution of category predictions, domain-specific knowledge, and general knowledge to balance general and domain-specific knowledge. **Figure 3** shows the pipeline of our method.

#### 3.2.1 Uncertainty score based on category prediction distribution

For CNN and ViT frameworks, we estimate the uncertainty score of test samples using the distribution of logits from the output categories of the fine-tuned model’s classification head. We refer to this branch as the uncertainty score based on category prediction distribution, denoted by *S_CPD_*, as shown in **Figure 3**. Please note that we allow for further processing of the logits, as is done in methods based on energy Liu et al. (2020). Take the max-logits Basart et al. (2022) as an example, for a test sample *x_i_*, the uncertainty score based on category prediction distribution is formalized as:


(10) $$S_{CPD}\left(x_{i}\right)=\text{Norm}\left(-\text{max }(\frac{z_{i}/T}{\sum_{i=1}^{K}z_{i}/T}\right)),$$

where *z_i_* denotes the logits of known class *i*, and *T* denotes the temperature scaling factor. Energy-based and MCM-based uncertainty scores are illustrated in Equations 7, 8.

#### 3.2.2 Uncertainty score based on domain-specific knowledge

The classification head retains only information directly relevant to making the classification decision, ignoring other information. In contrast, the final layer of the feature extractor typically contains a highly abstract and compressed representation of the original data, preserving more visual information. To effectively utilize the visual information in the feature extractor, we draw on the concept-matching approach and propose visual information matching. Specifically, given the fine-tuned model *M_ft_*, we compute the features *M_ft_* (*X_t_*) for all training set samples *X_t_*. For a test sample *x_i_*, we compute the feature *M_ft_* (*x_i_*). Then, we normalize *g_i_* and *f_t_* and calculate the maximum similarity, taking the negative value as the uncertainty score. Finally, the uncertainty scores for all samples are normalized to the [0,1] interval. Since fine-tuned models generally focus better on the domain-specific knowledge of downstream tasks, we refer to this as the uncertainty score based on domain-specific knowledge, denoted by *S_DSK_* (as shown in **Figure 3**). For a test sample *x_i_*, the uncertainty score based on domain-specific knowledge can be formalized as:


(11) $$S_{DSK}\left(x_{i}\right)=\text{Norm}\left(-\text{max }(similarity\left(M_{ft}\left(X_{t}\right),M_{ft}\left(x_{i}\right)\right)\right)).$$

#### 3.2.3 Uncertainty score based on general knowledge

We argue that the general knowledge extracted through the original pre-trained model can enhance anomaly detection performance. Therefore, given the original pre-trained model *M_pt_*, we compute the features *M_pt_* (*X_t_*) for all training set samples *X_t_*. For a test sample *x_i_*, we compute the feature *M_pt_* (*x_i_*). Then, we normalize *M_pt_* (*x_i_*) and *M_pt_* (*X_t_*) and calculate the maximum similarity, taking the negative value as the uncertainty score. In this study, we use cosine similarity to compute feature similarity. Similar to the domain-specific feature-matching process, the uncertainty scores for all samples are normalized to the [0,1] interval. For a test sample *x_i_*, the uncertainty score based on general knowledge can be formalized as:


(12) SGK(xi)=Norm(−max(similarity(Mpt(Xt),Mpt(xi)))),

Finally, given a text sample *x_i_*, the final ensemble uncertainty scores can be formalized as **Equation 13**:


(13) $$S\left(x_{i}\right)=\{\begin{array}{ll} \left(S_{CPD}\left(x_{i}\right)+S_{DSK}\left(x_{i}\right)+S_{GK}\left(x_{i}\right)\right)/3 & for\;CNNs\;or\;ViTs\\ \left(S_{DSK}\left(x_{i}\right)+S_{GK}\left(x_{i}\right)\right)/2 & for\;VLMs \end{array}.$$

We use three types of knowledge integration for purely visual frameworks like CNN and ViT. For VLM, we only utilize feature similarity scores from the visual branch for evaluation. The detailed implementation of this method can be found in **Algorithm 1**.

Algorithm 1 **Knowledge-Ensemble-Based uncertainty scoring.**

**Input:** training set *X^train^
*, test set *X^test^
*, fine-tuned model *M_ft_
*, pre-trained model *M_pt_
*, logits *z_i_
*from *M_ft_
*, temperature *T*
**Output:** uncertainty score *S*(*x_i_
*)
**for** each test sample *x_i_
*for each test sample *x_i_
*∈ *X^test^
* **do**
1. C**ompute** *S_CPD_
*(*x_i_
*):
For CNN or ViT, use the output logits from the linear classification head after the image encoder.
For VLM, use the similarity score between the image and textual embeddings.
2. C**ompute** *S_DSK_
*(*x_i_
*):
Extract features *f_i_
*= *M_ft_
*(*x_i_
*) and *F_t_
*= *M_ft_
*(*X^train^
*). Note that the “features” here refer exclusively to those extracted by the image encoder, excluding features from the text encoder.
Normalize extracted features, then compute cosine similarity
Compute *S_DSK_
*(*x_i_
*) according to Equation 11
3. **Compute** *S_GK_
*(*x_i_
*):
Extract features *M_pt_
*(*x_i_
*) and *M_pt_
*(*X^train^
*). Note that the “features” here refer exclusively to those extracted by the image encoder, excluding features from the text encoder.
Normalize extracted features, then compute cosine similarity
Compute *S_GK_
*(*x_i_
*) according to Equation 124. **Final Uncertainty Score:**
Fuse scores according to Equation 13 to obtain *S*(*x_i_
*)
**end for**

## 4 Experiments

### 4.1 Implementation details

For the convolutional neural network and vision transformer frameworks, we used ConvNeXt-base Liu et al. (2022) and ViT-base-patch16–224 Dosovitskiy et al. (2020) as backbone networks. These models have nearly equivalent parameters (87.57M in ConvNeXt-base vs. 85.80M in ViT-base-patch16-224) and were both pre-trained on the ImageNet-21k dataset Deng et al. (2009). For the vision-language model framework, we adopted CLIP Radford et al. (2021) with the CLIP-ViT-B/16 backbone.

For each architecture (CNN, ViT, and VLM), we fine-tune a backbone model using supervision from known classes only. Specifically, for CNN and ViT models, we adopt a standard linear classifier head and optimize using cross-entropy loss. For vision-language models (VLMs) such as CoCoOp, we fine-tune the learnable text prompts while keeping the vision encoder frozen, and the model is trained using a contrastive loss between image and text embeddings, following the CLIP training paradigm. Additionally, for the fine-tuning methods related to CNN and ViT architectures, we adopted the publicly available codebase provided by Jia et al. (2022) at https://github.com/KMnP/vpt, which includes implementations of adapter tuning and prompt-based tuning. For the VLM-related fine-tuning methods, we utilized the codebase released by Shen et al. (2024) at https://github.com/sIncerass/MVLPT/tree/main.

All models were optimized using the AdamW optimizer with a weight decay of 0.01. The initial learning rate was set to 0.0001 for full fine-tuning (FFT) and 0.001 for parameter-efficient tuning methods (e.g., VPT, VAT). The learning rate was linearly warmed up over the first 10 epochs and then decayed using a cosine scheduler. Training was performed for 100 epochs with a batch size of 128, and early stopping was applied based on the best validation accuracy. All experiments were conducted using PyTorch v1.10.0 on a single Nvidia RTX 4090 GPU.

After fine-tuning, we evaluate test samples using three score-level uncertainty measures: *S*_CPD_ (category prediction distribution from classification logits), *S*_DSK_ (domain-specific knowledge via feature similarity from the fine-tuned model), and *S*_GK_ (general knowledge via feature similarity from the frozen pre-trained model). The final anomaly score is computed as a non-parametric ensemble of these three components (see Equation 13). The final decision on whether a sample belongs to a known or unknown class is made based on a thresholding strategy applied to the ensemble score, as described in Equation 5.

Our enhancement method does not require additional training as long as pre-trained and fine-tuned models are available. The classification stage uses cross-entropy loss, while cosine similarity loss is used for the similarity-based scoring components, depending on the specific configuration.

### 4.2 Dataset splits

We utilize the Plant Village dataset Hughes and Salathé (2015) to construct a few-shot anomaly detection benchmark. Then, we proposed an enhancement method based on this benchmark. Plant Village Dataset Hughes and Salathé (2015) is a plant disease dataset containing 54,309 images, covering a total of 38 categories of both healthy and diseased leaf samples. Among these 38 categories, there are samples of 12 species of healthy leaves and 26 diseased leaves. The disease types include fungi, bacteria, oomycetes, viruses, and mites. In our study, we use the images of 12 types of healthy leaves as known categories to build the training dataset, and the remaining 26 disease categories are considered as unknown classes, included in the test dataset. We validated our method’s effectiveness using this partitioning strategy The dataset split information of the Plant Village dataset is shown in **Table 1**.

**Table 1:** Training and testing splits for the Plant Village dataset.

Dataset split	Known classes		Unknown classes	
	Classes	Images	Classes	Images
Training	12	12062	–	–
Testing	12	3021	26	39220
Known Class Names	Cherry healthy leaf, Tomato healthy leaf, Grape healthy leaf, Raspberry healthy leaf, Apple healthy leaf, Blueberry healthy leaf, Soybean healthy leaf, Strawberry healthy leaf, Peach healthy leaf, Potato healthy leaf, Corn healthy leaf, Bell Pepper healthy leaf			

The 12 known classes are selected from 12 healthy categories in the Plant Village dataset, such as healthy apple leaves.

We extended our method to more datasets to verify its effectiveness, including: Cotton Disease Dataset Dhamodharan (2023). This dataset contains approximately 4,800 images covering six categories: five cotton leaf disease types and one healthy category. The dataset maintains a balanced distribution with around 800 images per category. In our experiments, we construct different settings with 2–4 known classes and 2 unknown classes.

Mango Leaf Disease Dataset Ahmed et al. (2023). This dataset comprises 4,000 images of mango leaves across eight categories: seven disease types and one healthy category. The images evenly distributed with approximately 500 images per category. We use different splits with 2–6 known categories and 2 unknown categories for evaluation.

Strawberry Disease Dataset Afzaal et al. (2021). This dataset includes 2,500 images representing various stages of strawberry leaf diseases and healthy leaves. To ensure consistency across benchmarks, images of healthy strawberry leaves from the PlantVillage dataset Hughes and Salathé (2015) are added. This updated dataset comprises 2,974 images of strawberry leaves across eight categories: seven disease types and one healthy category. We configure 2–6 categories as known and 2 categories as unknown for anomaly detection evaluation.

Tomato Disease Dataset Hughes and Salathé (2015). A subset of the PlantVillage dataset, this collection focuses on tomato leaf conditions, containing 10 categories in total — 9 disease types and 1 healthy category. It exhibits class imbalance, with per-class image counts ranging from 300 to over 5,000. In our settings, we select 2–7 categories as known and 3 categories as unknown. More detailed category assignment can be found in **Table 2**.

**Table 2:** Division of known and unknown classes for other four dataset.

Class id	Cotton classes name	Mango classes name	Strawberry classes name	Tomato classes name
1	Healthy	Healthy	Healthy	Healthy
2	Powdery mildew	Sooty mould	Powdery mildew leaf	Early blight
3	Target spot	Anthracnose	Anthracnose fruit rot	Leaf mold
4	Aphids	Powdery mildew	Leaf spot	Spider mites
5	Bacterial blight	Bacterial canker	Powdery mildew fruit	Septoria leaf spot
6	Army worm	DieBack	Blossom blight	Mosaic virus
7	–	Cutting weevil	Angular leaf spot	Bacterial spot
8	–	Gall midge	Gray mold	Late blight
9	–	–	–	Yellow leaf curl virus
10	–	–	–	Target spot
Below is the division of known (K) and unknown (U) classes for each dataset.				
Exp. no.	Cotton (K/U)	Mango (K/U)	Strawberry (K/U)	Tomato (K/U)
1	1,2/5,6	1,2/7,8	1,2/7,8	1,2/8,9,10
2	1,2,3/5,6	1,2,3/7,8	1,2,3/7,8	123/8,9,10
3	1,2,3,4/5,6	1,2,3,4/7,8	1,2,3,4/78	1,2,3,4/8,9,10
4	–	1,2,3,4,5/7,8	1,2,3,4,5/7,8	1,2,3,4,5/8,9,10
5	–	1,2,3,4,5,6/7,8	1,2,3,4,5,6/7,8	1,2,3,4,5,6/8,9,10
6	–	–	–	1,2,3,4,5,6,7/8,9,10

K denotes known classes, and U denotes unknown classes.

To enhance reproducibility and clarity, we have added details regarding how the class splits are configured. For example, in the first experiment on the Tomato dataset, categories 1 and 2 are assigned as known classes, while categories 8, 9, and 10 are treated as unknown classes. Specifically, Healthy and Early Blight are treated as known categories, whereas Late Blight, Yellow Leaf Curl Virus, and Target Spot are treated as unknown. The model is required to perform classification among the known categories, and the classification accuracy on these is evaluated using standard accuracy metrics. During inference, when encountering samples from the three unknown categories, the model should ideally reject these samples. If the model classifies Early Blight as unknown, or misclassifies Late Blight as Early Blight, these are considered incorrect predictions. Such misclassifications negatively impact the AUROC score and increase the FPR@TPR95 score.

### 4.3 Main results

In this section, we present the main results of our study, as summarized in **Tables 3**, **4**. The results are analyzed from three perspectives: training frameworks, fine-tuning paradigms, sample sizes, and anomaly detection methods. Note that “Ours” in **Tables 3**, **4** refers to the deployment of our proposed method. When using our approach, each model requires both a fine-tuned and a frozen version. In contrast, the other methods rely solely on the fine-tuned model.

**Table 3:** Plant disease anomaly detection benchmark and our enhanced method.

Methods		FPR@TPR95↓/AUROC↑ at different shots				
*CNN-based*	Promots	2-shot	4-shot	8-shot	16-shot	All-shot
*FFT_Energy_*	–	89.59/56.54	74.86/75.70	50.20/88.22	68.37/74.24	7.91/96.72
*FFT_Energy_* (*Ours*)	–	53.24/83.30	23.60/95.77	10.71/97.47	21.83/94.02	1.21/99.72
*FFT*_*Max*−*Logits*_	–	89.23/56.53	75.40/75.66	51.70/88.19	67.92/74.37	7.91/96.72
*FFT*_*Max*−*Logits*_(*Ours*)	–	53.05/83.29	23.87/95.73	11.09/97.44	21.51/94.14	1.21/99.72
*VAT_Energy_*	–	98.11/44.86	84.97/66.97	92.00/53.84	68.49/71.99	16.20/95.86
*VAT_Energy_* (*Ours*)	–	38.71/91.50	24.31/95.28	10.10/97.75	10.81/97.66	1.75/99.60
*VAT*_*Max*−*Logits*_	–	98.03/44.91	85.21/66.59	91.62/54.79	68.63/72.11	16.08/95.89
*VAT*_*Max*−*Logits*_(*Ours*)	–	38.80/91.50	24.33/95.29	10.01/97.74	10.78/97.66	1.74/99.60
*VPT_Energy_*	–	89.59/56.54	74.86/75.70	50.20/88.22	68.37/74.24	7.91/96.72
*VPT_Energy_* (*Ours*)	–	26.27/94.83	27.42/93.75	20.64/94.18	10.67/97.55	1.78/99.64
*VPT*_*Max*−*Logits*_	–	89.23/56.53	75.40/75.66	51.70/88.19	67.92/74.37	7.91/96.72
*VPT*_*Max*−*Logits*_(*Ours*)	–	26.27/94.83	27.42/93.75	20.57/94.20	10.67/97.55	1.70/99.66
*ViT-based*	Promots	2-shot	4-shot	8-shot	16-shot	All-shot
*FFT_Energy_*	–	72.42/78.32	68.10/83.26	26.47/94.93	21.87/95.65	6.14/98.62
*FFT_Energy_* (*Ours*)	–	34.39/93.13	27.11/95.40	10.36/98.06	7.84/98.33	1.68/99.62
*FFT*_*Max*−*Logits*_	–	72.59/78.20	65.58/83.60	28.62/94.70	21.78/95.59	6.13/98.61
*FFT*_*Max*−*Logits*_(*Ours*)	–	34.82/92.87	28.07/95.33	10.91/97.99	7.96/98.30	1.66/99.62
*VAT_Energy_*	–	48.31/84.35	70.39/83.50	49.80/86.80	43.43/90.32	6.25/98.44
*VAT_Energy_* (*Ours*)	–	26.06/94.60	36.15/93.34	13.68/96.94	16.69/96.36	2.28/99.55
*VAT*_*Max*−*Logits*_	–	53.93/83.04	73.57/83.21	49.58/86.79	44.10/90.35	6.25/98.44
*VAT*_*Max*−*Logits*_(*Ours*)	–	26.25/94.38	37.77/93.16	13.79/96.97	17.90/96.31	2.25/99.56
*VPT_Energy_*	–	54.74/88.62	61.26/87.89	22.49/95.91	25.81/94.78	8.82/98.36
*VPT_Energy_* (*Ours*)	–	30.73/94.41	29.23/94.37	11.06/97.96	13.30/97.23	2.11/99.54
*VPT*_*Max*−*Logits*_	–	58.75/87.55	56.10/88.46	24.34/95.68	26.44/94.64	8.81/98.35
*VPT*_*Max*−*Logits*_(*Ours*)	–	29.79/94.12	29.43/94.31	11.90/97.85	13.36/97.20	1.99/99.57
*VLM-based*	Promots	2-shot	4-shot	8-shot	16-shot	All-shot
*CoO_pMCM_*	LP + [CLS]	93.50/54.40	86.93/56.91	89.32/60.09	81.94/66.87	68.97/75.28
*CoO_pMCM_* (*Ours*)	LP + [CLS]	41.51/91.64	35.16/92.62	29.36/94.34	23.50/95.63	9.77/98.29
*CoCoO_pMCM_*	LP + [CLS]	91.81/60.92	85.39/63.99	77.37/76.63	64.89/81.20	40.08/88.61
*CoCoO_pMCM_* (*Ours*)	LP + [CLS]	41.51/91.64	35.16/92.62	29.36/94.34	23.50/95.63	9.77/98.29
*VPT_MCM_*	FP + [CLS]	85.01/68.24	72.90/73.66	61.74/84.61	51.55/84.20	15.67/96.23
*VPT_MCM_* (*Ours*)	FP + [CLS]	38.17/91.13	21.68/94.94	15.12/97.01	9.36/97.83	0.53/99.87
*VLPT_MCM_*	LP + [CLS]	80.60/72.00	73.48/72.64	63.88/80.34	43.88/87.77	15.38/96.50
*VLPT_MCM_* (*Ours*)	LP + [CLS]	27.98/93.81	16.17/96.16	9.05/98.16	7.05/98.41	0.71/99.85

LP and FP denote a learnable prompt and fixed prompt where the fixed prompt is ‘This is a photo of a ‘. [CLS] denotes known class names. We present the results of deploying our method across various training frameworks, fine-tuning paradigms, shots, and anomaly detection methods.

**Table 4:** Accuracy of known classes in Plant disease anomaly detection Benchmark.

Methods	Prompts	Accuracy of known classes ↑ at different shots				
*CNN-based*		2-shot	4-shot	8-shot	16-shot	All-shot
*FFT*	–	77.99	90.50	96.13	98.51	100.00
*VAT*	–	45.55	42.20	59.68	84.57	100.00
*VPT*	–	88.58	96.13	97.91	99.01	100.00
*ViT-based*		2-shot	4-shot	8-shot	16-shot	All-shot
*FFT*	–	81.76	89.57	93.15	95.70	100.00
*VAT*	–	73.55	87.32	97.29	97.48	100.00
*VPT*	–	91.56	95.27	98.41	98.64	100.00
*VLM-based*		2-shot	4-shot	8-shot	16-shot	All-shot
*CoOp*	learnable prompt + [CLS]	78.07	85.83	92.73	96.33	99.20
*CoCoOp*	learnable prompt + [CLS]	74.13	79.60	92.03	95.83	99.20
*VPT*	This is a photo of a + [CLS]	86.90	92.93	95.77	98.60	100.00
*VLPT*	learnable prompt + [CLS]	84.23	90.87	96.00	98.07	99.99

[CLS] denotes known class names. We present the results of deploying our method across various training frameworks, fine-tuning paradigms, and shots.

#### 4.3.1 Training frameworks

The benchmark includes three types of training frameworks: CNN-based, ViT-based, and VLM-based. For CNN-based models, the baseline methods demonstrated decent performance under all-shot settings, with AUROC values of 96.72% for *FFT_Energy_* and 95.89% for *V AT_Max–Logits_*. However, these methods struggled in low-shot settings, such as 2-shot, where AUROC scores dropped to 56.54% and 44.91%, respectively. In contrast, our enhanced methods significantly improved performance. For example, *FFT_Energy_* (*Ours*) achieved an AUROC of 83.30% in the 2-shot setting, a remarkable improvement of nearly 27% over the baseline. Similarly, *V AT_Energy_* (*Ours*) improved the 2-shot AUROC from 44.86% to 91.50%. Across all-shot settings, our enhancements consistently achieved near-perfect AUROC scores, such as 99.72% for *FFT_Energy_* (*Ours*).

For ViT-based models, baseline methods exhibited weaker performance under low-shot settings. For instance, *FFT_Energy_* achieved an AUROC of 78.32% in the 2-shot setting. However, our enhanced method *FFT_Energy_* (*Ours*) dramatically improved this to 93.13%, an improvement of over 14%. Similarly, *V AT_Energy_* (*Ours*) outperformed its baseline counterpart in the 2-shot setting, achieving an AUROC of 94.60% compared to 84.35%. This trend was consistent across different shot counts, with the enhancements narrowing the performance gap between low-shot and all-shot settings.

The VLM-based models showed the most dramatic improvements with our enhancements. For example, in the 2-shot setting, the baseline *CoOp_MCM_* achieved an AUROC of only 54.40%, while our enhanced *CoOp_MCM_* (*Ours*) reached 91.64%, an improvement of over 37%. Similarly, *V LPT_MCM_* (*Ours*) achieved 93.81% in the 2-shot setting, compared to just 72.00% for the baseline. These results underscore the ability of our method to effectively leverage multi-modal architectures for anomaly detection.

#### 4.3.2 Sample sizes

The evaluation across different sample sizes (2-shot, 4-shot, 8-shot, 16-shot, and all-shot) revealed consistent trends.

In the 2-shot setting, baseline methods generally struggled. For example, *FFT_Energy_* in the CNN-based framework achieved an AUROC of 56.54%, while our enhanced method *FFT_Energy_* (*Ours*) improved this to 83.30%. Similarly, for the VLM-based framework, *V LPT_MCM_* achieved 72.00%, whereas *V LPT_MCM_* (*Ours*) reached an impressive 93.81%.

As the sample size increased, both baseline and enhanced methods showed improved performance. For instance, in the CNN-based framework, *FFT_Energy_* (*Ours*) achieved an AUROC of 95.77% at 4shot and 99.72% at all-shot, maintaining robust performance throughout. In the VLM-based framework, *V LPT_MCM_* (*Ours*) achieved 98.16% with only 8-shot, demonstrating capability in few-shot learning. When all available data is used for training, our method attains almost perfect AUROC scores close to or exceeding 99.5%, which indicates that the model can correctly detect almost all anomalous samples.

As for the accuracy of known classes, increasing the number of training samples per class leads to substantial improvements in classification accuracy across all models. As shown in **Table 4**, most frameworks reach over 95% accuracy by the 8-shot setting and converge to nearly 100% in the all-shot configuration. This pattern confirms that the models are capable of quickly learning the known class decision boundaries with relatively few examples. It also provides reassurance that errors observed in anomaly detection are not due to poor discrimination among known categories, but rather the inherent difficulty of detecting unknowns.

While the overall trend aligns with the expectation that performance improves with more data, minor inconsistencies—such as 8-shot occasionally outperforming 16-shot in CNN-based settings—can be observed. We attribute these fluctuations to the randomness of few-shot data splits and the instability of training CNNs under low-data regimes. These variations are within the expected range in few-shot learning scenarios and do not undermine the overall upward trend.

#### 4.3.3 Anomaly detection methods

We evaluated multiple anomaly detection approaches, including energy-based and max-logits-based methods. In general, energy-based methods achieved results comparable to their max-logits counterparts. For example, in the CNN-based framework, *FFT_Energy_* achieved an AUROC of 96.72% in the all-shot setting, identical to that of *FFT_Max_*_–_ *_Logits_*. Similarly, in the ViT-based framework under the 2-shot setting, *FFT_Energy_* (Ours) reached 93.13% AUROC, while *FFT_Max–Logits_* (Ours) achieved 92.87%.

Across CNN-, ViT-, and VLM-based frameworks, our enhanced multi-modal methods consistently demonstrated substantial improvements. For instance, *CoO_pMCM_* (Ours) achieved an AUROC of 91.64% in the 2-shot setting, significantly outperforming the baseline *CoO_pMCM_* at 54.40%. Similarly, *V LPT_MCM_* (Ours) reached 99.85% in the all-shot setting, surpassing the baseline score of 96.50%.

Overall, our proposed method consistently improved the performance of baseline anomaly detection approaches across different model architectures, fine-tuning paradigms, and few-shot configurations. These results indicate that our method generalizes effectively and enhances robustness in low-shot scenarios, providing a solid foundation for advancing anomaly detection in plant disease recognition tasks.

### 4.4 Ablation study

**Figure 4:**
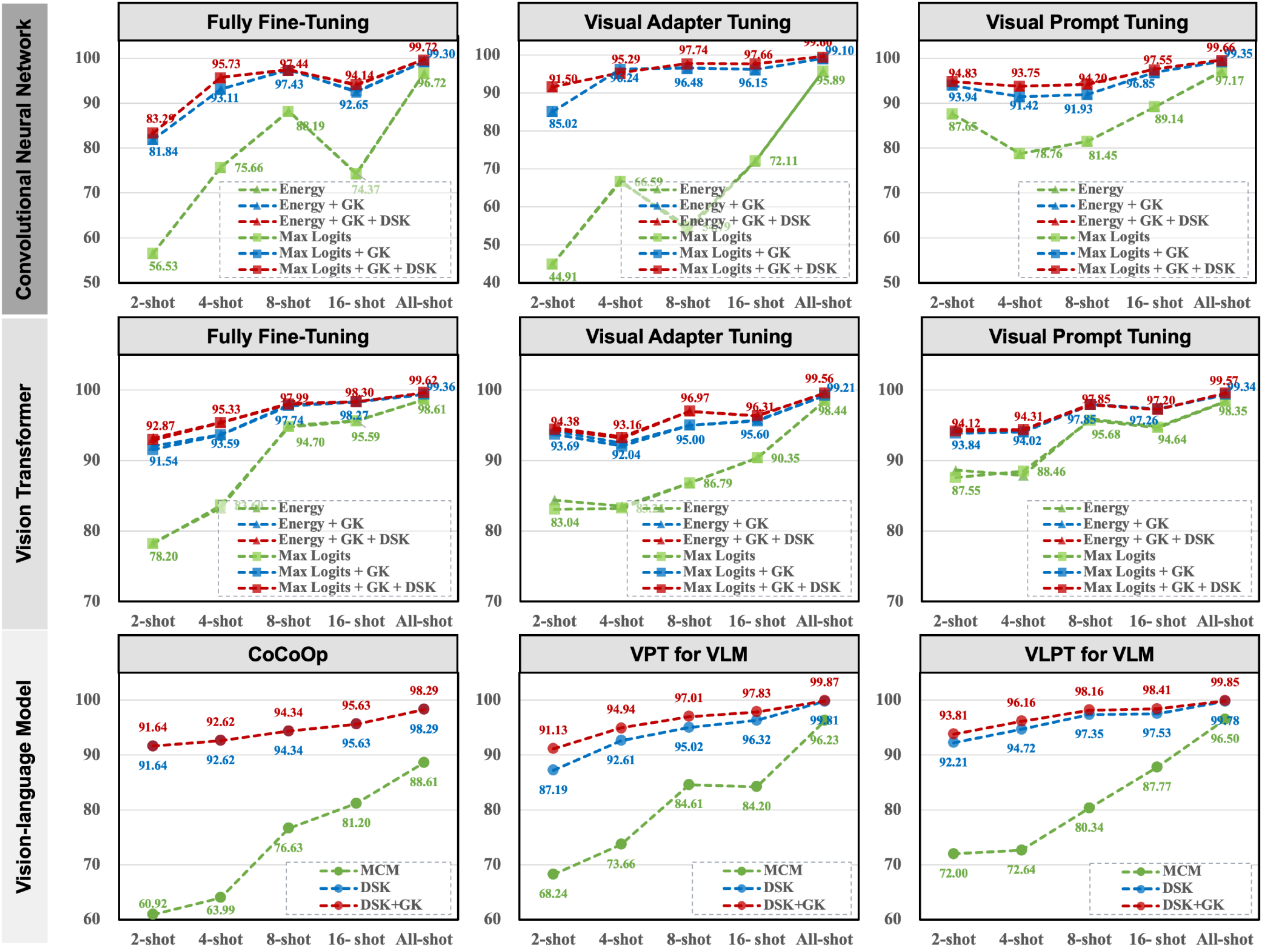
Ablation results on the Plant Village dataset under different training frameworks, fine-tuning paradigms, shots, and anomaly detection methods.

We present the results of our ablation experiments in **Figure 4**, which evaluate the contributions of different components of our method under various training frameworks, fine-tuning paradigms, and shot configurations. The results demonstrate that integrating general knowledge from pre-trained models and combining it with domain-specific knowledge significantly improves anomaly detection performance, particularly in few-shot settings.

The baseline method, represented by the green line, shows limited performance across all frameworks, especially in low-shot scenarios. For instance, in the fully fine-tuned CNN-based framework, the AUROC starts at 56.53% in the 2-shot setting and only reaches 96.72% in the all-shot setting, highlighting the model’s inability to generalize with limited data. By integrating general knowledge (blue line), the performance improves substantially, with AUROC increasing to 93.69% in the Vision Transformer framework (2-shot, visual adapter tuning) and 93.81% in the VLM-based VLPT model (2-shot). This demonstrates the importance of leveraging the pre-trained model’s general knowledge to handle few-shot scenarios effectively.

Further, the ensemble of general and domain-specific knowledge (red line) achieves the most significant improvements, consistently outperforming the baseline and general knowledge integration across all frameworks and fine-tuning paradigms. For example, in the Vision Transformer framework with fully fine-tuned models, the AUROC improves from 78.20% (baseline) to 94.95% (ensemble) in the 2-shot setting. Similarly, in the VLM-based VLPT model, the AUROC reaches 99.85% in the all-shot setting, demonstrating near-perfect performance. Importantly, our method reduces the discrepancies in detection performance across fine-tuning paradigms. For instance, in the CNN-based framework, visual adapter tuning achieves an AUROC of 97.14% in the all-shot setting, closely aligning with the fully fine-tuned paradigm at 99.72%.

Although the paradigm of parameter-efficient fine-tuning has preserved most of the general knowledge in the original pre-trained model, the explicit integration of this general knowledge can still further enhance anomaly detection performance. For instance, in VLM cases, we observe a slight performance improvement. In summary, the ablation study highlights that our method not only improves performance in few-shot settings but also ensures consistent and robust results across different training frameworks and fine-tuning paradigms.

### 4.5 Generalizability

To assess the generalization capability of our method beyond clean benchmark datasets, we conducted additional experiments on a real-world Cotton Disease dataset, which was collected under diverse field conditions, including natural illumination variations, complex backgrounds, and non-uniform image quality. This dataset presents a more challenging scenario compared to studio-collected datasets such as PlantVillage.

As shown in **Table 5**, our method consistently improves the performance of baseline methods across various fine-tuning types (FFT, VAT, VPT) and few-shot settings (from 2-shot to all-shot). The improvements are particularly pronounced under limited-sample conditions. For example, in the 4-shot setting, *VAT_Max-Logits_(Ours)* reduces the FPR@TPR95 from 81.71% to 58.87%. Moreover, as the number of samples increases, the model’s ability to distinguish unknown samples improves significantly. Under the VPT prompt, for instance, our method (*VPT_Max-Logits_(Ours)*) achieves an FPR@TPR95 of 12.24% and AUROC of 97.15% in the all-shot setting, which is highly competitive given the variability of field-acquired imagery. Similarly, in both FFT and VAT settings, our method achieves the best or second-best performance in the 8-shot and all-shot settings, demonstrating robustness in data-scarce scenarios.

**Table 5:** Generalization results.

Methods	Prompts	FPR@TPR95↓/AUROC↑ at different shots			
ViT-based	2-shot	4-shot	8-shot	16-shot	All-shot
*FFT_Energy_*	81.71/77.04	73.73/77.65	77.58/81.53	65.34/88.81	24.76/95.52
*FFT_Energy_* (*Ours*)	76.34/79.08	69.92/81.88	61.21/86.12	51.44/89.83	16.23/97.55
*FFT*_*Max*−*Logits*_	80.47/75.74	73.59/76.65	77.27/81.55	65.75/88.66	24.48/95.54
*FFT*_*Max*−*Logits*_(*Ours*)	73.63/78.41	70.45/78.36	67.54/85.03	52.68/89.08	15.27/97.59
*VAT_Energy_*	91.06/70.01	81.98/78.02	66.99/78.13	63.41/83.64	14.72/97.51
*VAT_Energy_* (*Ours*)	83.49/76.99	64.51/80.05	57.22/83.59	51.44/86.93	12.52/97.72
*VAT*_*Max*−*Logits*_	90.78/69.94	81.71/78.14	64.10/78.60	63.55/84.10	13.20/97.69
*VAT*_*Max*−*Logits*_(*Ours*)	77.99/77.54	58.87/80.12	59.70/83.80	53.65/87.15	12.38/97.77
*VPTEnergy*	96.29/60.13	79.78/76.67	68.23/80.24	51.44/85.51	13.76/96.77
*VPT_Energy_* (*Ours*)	77.30/77.52	62.31/82.72	67.40/81.40	51.17/87.65	12.24/96.98
*VPT*_*Max*−*Logits*_	94.77/61.61	85.01/77.95	66.16/81.06	51.86/85.71	13.20/96.83
*VPT*_*Max*−*Logits*_(*Ours*)	72.63/77.66	63.41/82.72	64.10/81.99	50.48/87.71	12.24/97.15

We evaluate the generalization capability of our method using the third experimental group on the Cotton dataset (refer to **Table 2**) with the standard ViT-base-patch16–224 model.

These results demonstrate that our approach not only performs well on standard benchmarks but also generalizes effectively to complex real-world agricultural data. However, we also observed that complex scenarios introduce significant challenges for anomaly detection. Under fow-shot settings, this may lead to performance degradation. For example, on the clean PlantVillage dataset, using just 2-shots can reduce FPR@TPR95 to 20%-30%(lower is better), whereas on the Cotton dataset, the same metric remains above 70% in all few-shot configurations. This limitation suggests that our method may require additional enhancement before being reliably deployed in practical agricultural systems.

### 4.6 Visualization of uncertainty distribution

**Figure 5:**
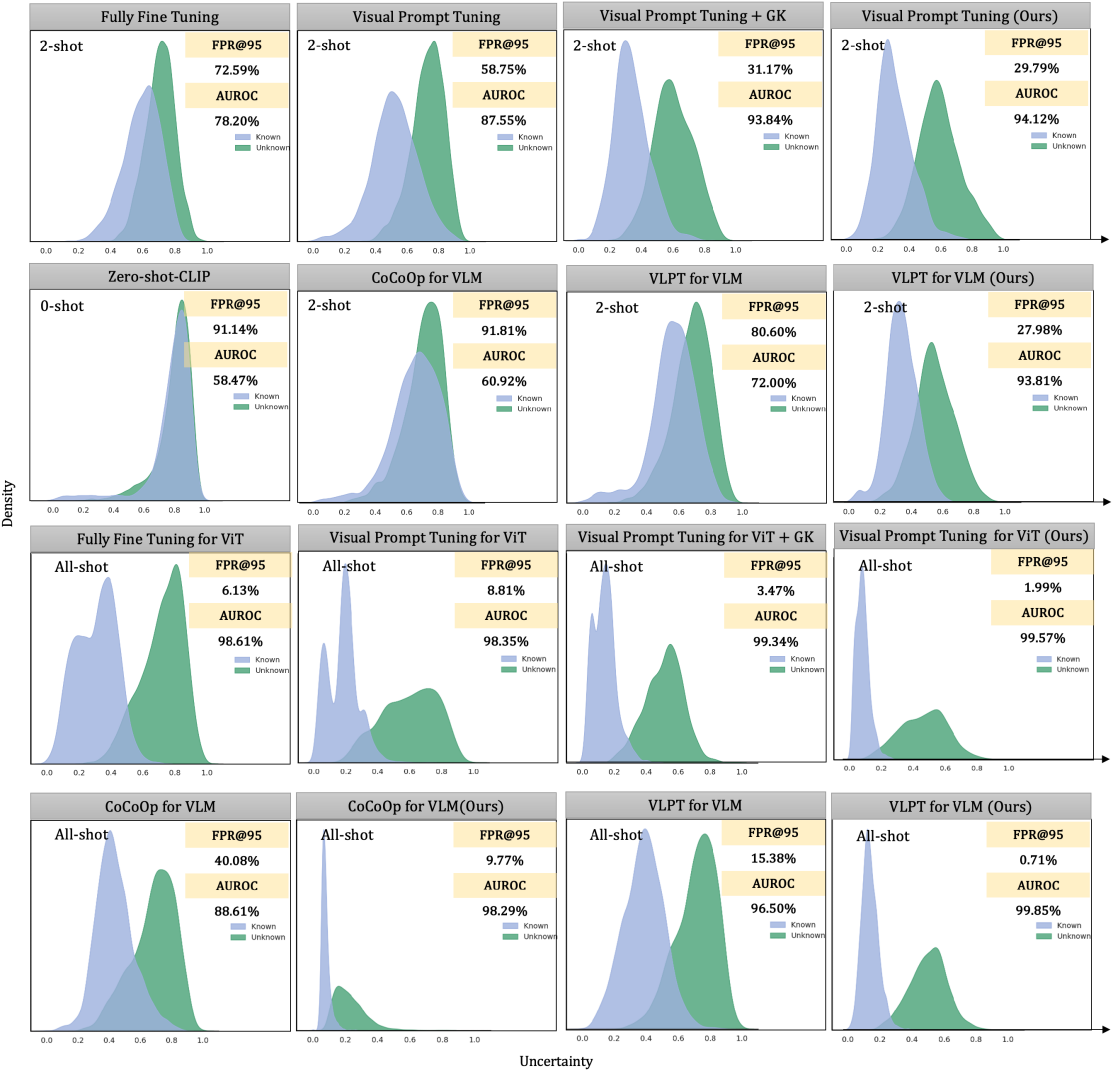
Comparing the uncertainty distributions among different frameworks, fine-tuning paradigms, and the deployment of the ensemble method. We fine-tuned on the Plant Village dataset in a 2-shot and all-shot settings and visualized these uncertainty distributions. For CoCoOp, we used the maximum concept matching to measure uncertainty. We calculated uncertainty using the maximum logits value for the single-modal visual pre-trained model.

To provide intuitive insight into how our knowledge integration method improves anomaly detection performance, we visualize the distributions of uncertainty scores for known and unknown samples across various training frameworks, fine-tuning paradigms, and ensemble configurations, as shown in **Figure 5**. These visualizations allow us to analyze how effectively different models separate known and unknown samples.

We use 12 healthy classes from the PlantVillage dataset as known categories and 26 diseased classes as unknowns. For zero-shot settings, vision-language models (VLMs) rely solely on text prompts constructed from class names. For CoOp and CoCoOp, we adopt their standard prompting strategies, appending class names to learnable tokens. In addition to zero-shot inference, we present comparisons under 2-shot and all-shot settings to examine performance across varying supervision levels.

The distributions reveal three important patterns:

1. Poor separability in zero-shot and baseline settings. Zero-shot CLIP achieves an AUROC of only 58.47%, indicating severe overlap between known and unknown distributions. Although CoCoOp adds prompt adaptability, it remains suboptimal in the 2-shot setting with an AUROC of 60.92%, suggesting difficulty in modeling fine-grained visual differences in plant diseases without stronger visual grounding.
2. Effectiveness of our knowledge integration method. When our method is applied (rightmost columns), the distributions exhibit significantly improved separability. For instance, under visual prompt tuning in the 2-shot setting, our method improves AUROC from 87.55% to 94.12% and reduces FPR@TPR95 from 58.75% to 29.79%. In the all-shot setting, the improvement is even more dramatic, achieving an AUROC of 99.57% and reducing FPR@TPR95 to 1.99%, clearly outperforming all baselines.
3. Empirical validation of the integration mechanism. Our method works by combining three complementary uncertainty perspectives: prediction confidence (*S_CPD_*), domain-specific feature similarity (*S_DSK_*), and general knowledge feature similarity (*S_GK_*). As seen in **Figure 5**, this multi-view fusion leads to tighter distributions for known classes and a wider margin between known and unknown distributions. This suggests that general knowledge from frozen models plays a critical role in calibrating over-confident predictions made by fine-tuned models, especially in low-shot settings.

In summary, the results validate the critical importance of preserving and effectively utilizing the general knowledge embedded in pre-trained models. While lightweight fine-tuning paradigms like visual prompt tuning partially retain this knowledge, our knowledge integration method amplifies it, leading to superior separability between known and unknown categories.

## 5 Discussion

### 5.1 Effect of ensemble strategies on VLMs

Recent research has shown that zero-shot language prompts in vision tasks and other lightweight fine-tuning methods have demonstrated potential for improving anomaly detection performance in natural language processing Ming et al. (2022); Ming and Li (2024). MCM Ming et al. (2022) matches the conceptual information of known categories with the visual features of test samples. However, designing effective language prompts for plant disease detection has proven to be more complex Dong et al. (2024a). For instance, using CoCoOp to generate adaptive context prompts for plant diseases reduced the FPR@TPR95 score to 40.08% in the all-shot setting, which is still much lower than expected. In comparison, we found that a single-modal visual pre-trained model achieved an FPR as low as 28.62% (max-logits in the ViT framework) in the 8-shot setting. Therefore, we argue that the visual symptoms of plant diseases are critical for distinguishing between known and unknown class samples. For fine-grained plant disease anomaly detection, a naive application of concept matching may introduce side effects, resulting in suboptimal performance.

**Figure 6:**
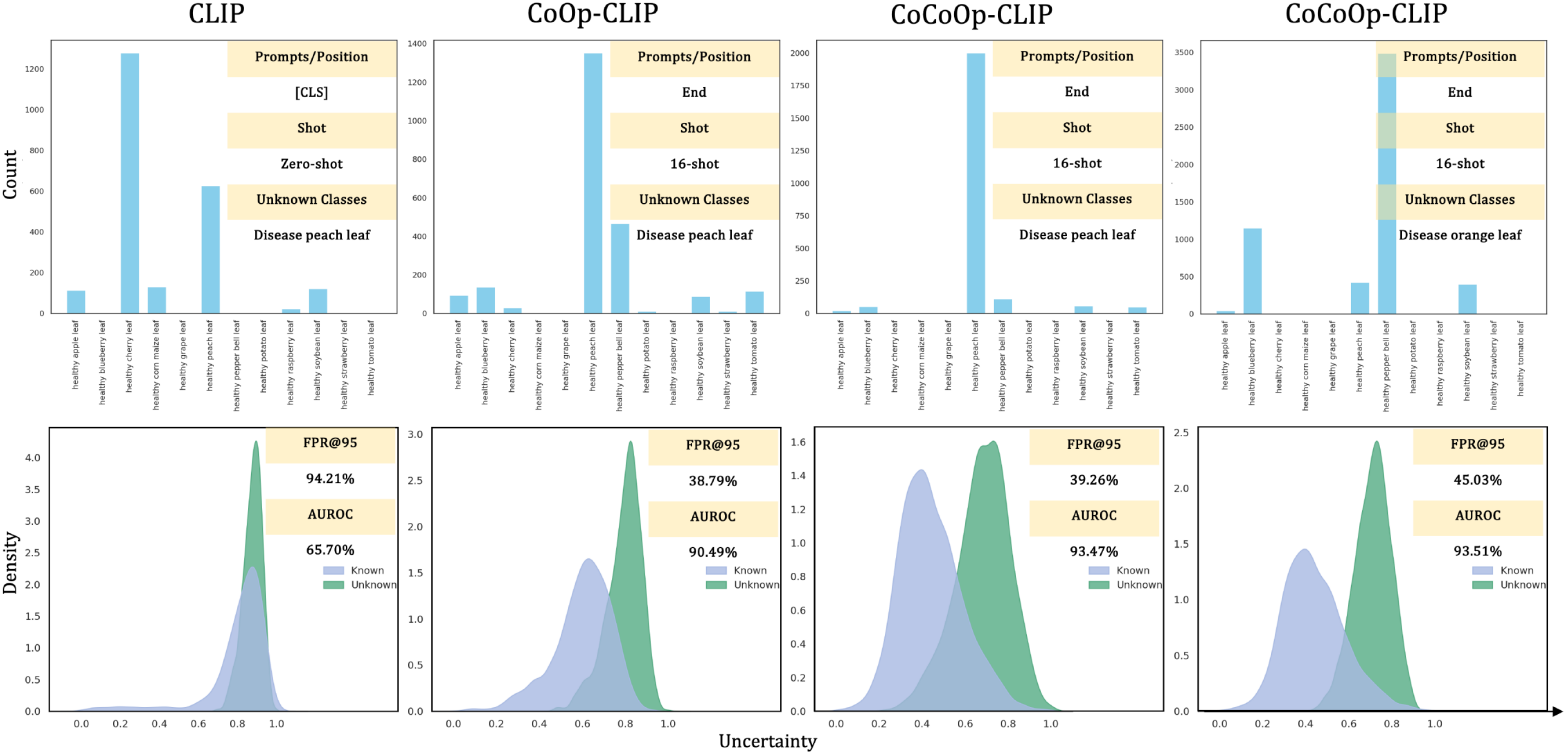
Visualization of predictions and uncertainty scores. We trained CLIP-ViT-B/16 on the Plant Village dataset and visualized the prediction results as well as the uncertainty distribution. Note that the uncertainty score is calculated by MCM.

To understand why performance is so much lower than expected, we visualize the distribution of uncertainty scores and the predicted category distribution in **Figure 6**. We first tested CLIP’s zero-shot anomaly detection performance using diseased peach leaves as unknown classes. For all the diseased peach leaf samples, approximately 50% were classified as healthy cherry leaves, and about 25% were classified as healthy peach leaves, which indicates the shortcoming of zero-shot CLIP in plant-related tasks. From the perspective of uncertainty scores, the distribution is concentrated around 0.9, suggesting that the model was fairly uncertain about both known and unknown classes. When we tested the model’s anomaly detection performance in a 16-shot setting using diseased peach leaves and orange leaves as unknown classes, CoCoOp significantly reduced the uncertainty of known class samples, which is shown in **Figure 6**. However, when testing the model with diseased peach leaves affected, the model confidently classifies most diseased peach leaves (more than 90% in CoCoOp) as healthy peach leaves. When testing with diseased orange leaves, the model still shows great uncertainty because the known classes do not include orange leaves. Therefore, we believe that vision-language models focus more on matching nouns and image features, neglecting adjectives, which severely impacts the VLMs’ performance in fine-grained identification tasks like plant disease anomaly detection.

Finally, we attempted to integrate MCM with our method, as we did with the CNN and ViT frameworks. However, while performance improved, it did not surpass the results obtained by directly using feature similarity to identify anomaly samples. Relevant results are provided in **Table 6**. Consequently, for VLM, we did not integrate category prediction distribution scores. Notably, our approach achieved an AUROC score of 91.64% in the 2-shot setting, significantly outperforming the baseline method [CoCoOp Ming et al. (2022)] fine-tuned on the entire dataset (88.61%). Furthermore, for visual prompt tuning and unified vision-language prompt tuning, we reduced the FPR@TPR95 to 0.53% and 0.71%, respectively, in the all-shot setting, which is remarkable.

**Table 6:** Results of the different ensemble strategies for the VLM framework.

Methods	Prompts	FPR@TPR95↓/AUROC↑ at different shots				
*CoCoOp*		2-shot	4-shot	8-shot	16-shot	All-shot
*MCM*	LP + [CLS]	91.81/60.92	85.39/63.99	77.37/76.63	64.89/81.20	40.08/88.61
*MCM* +*GK* +*DSK*	LP + [CLS]	47.95/85.44	39.09/88.35	30.15/92.73	23.11/94.72	7.94/98.25
*GK* +*DSK*(*Ours*)	LP + [CLS]	41.51/91.64	35.16/92.62	29.36/94.34	23.50/95.63	9.77/98.29
*VPT*		2-shot	4-shot	8-shot	16-shot	All-shot
*MCM*	FP + [CLS]	85.01/68.24	72.90/73.66	61.74/84.61	51.55/84.20	15.67/96.23
*MCM* +*GK* +*DSK*	FP + [CLS]	58.79/82.86	42.19/88.99	31.71/92.96	23.12/94.15	2.26/99.56
*GK* +*DSK*(*Ours*)	FP + [CLS]	38.17/91.13	21.68/94.94	15.12/97.01	9.36/97.83	0.53/99.87
*VLPT*		2-shot	4-shot	8-shot	16-shot	All-shot
*MCM*	LP + [CLS]	80.60/72.00	73.48/72.64	63.88/80.34	43.88/87.77	15.38/96.50
*MCM* +*GK* +*DSK*	LP + [CLS]	38.51/89.83	30.21/91.46	20.41/95.20	13.22/96.80	1.79/99.64
*GK* +*DSK*(*Ours*)	LP + [CLS]	27.98/93.81	16.17/96.16	9.05/98.16	7.05/98.41	0.71/99.85

LP and FP denote a learnable and fixed prompt where the fixed prompt is ‘This is a photo of a ‘. [CLS] denotes known class names. We present the results of deploying our method across various training frameworks, fine-tuning paradigms, and shots.

### 5.2 Validation of our method in open-set settings

**Figure 7:**
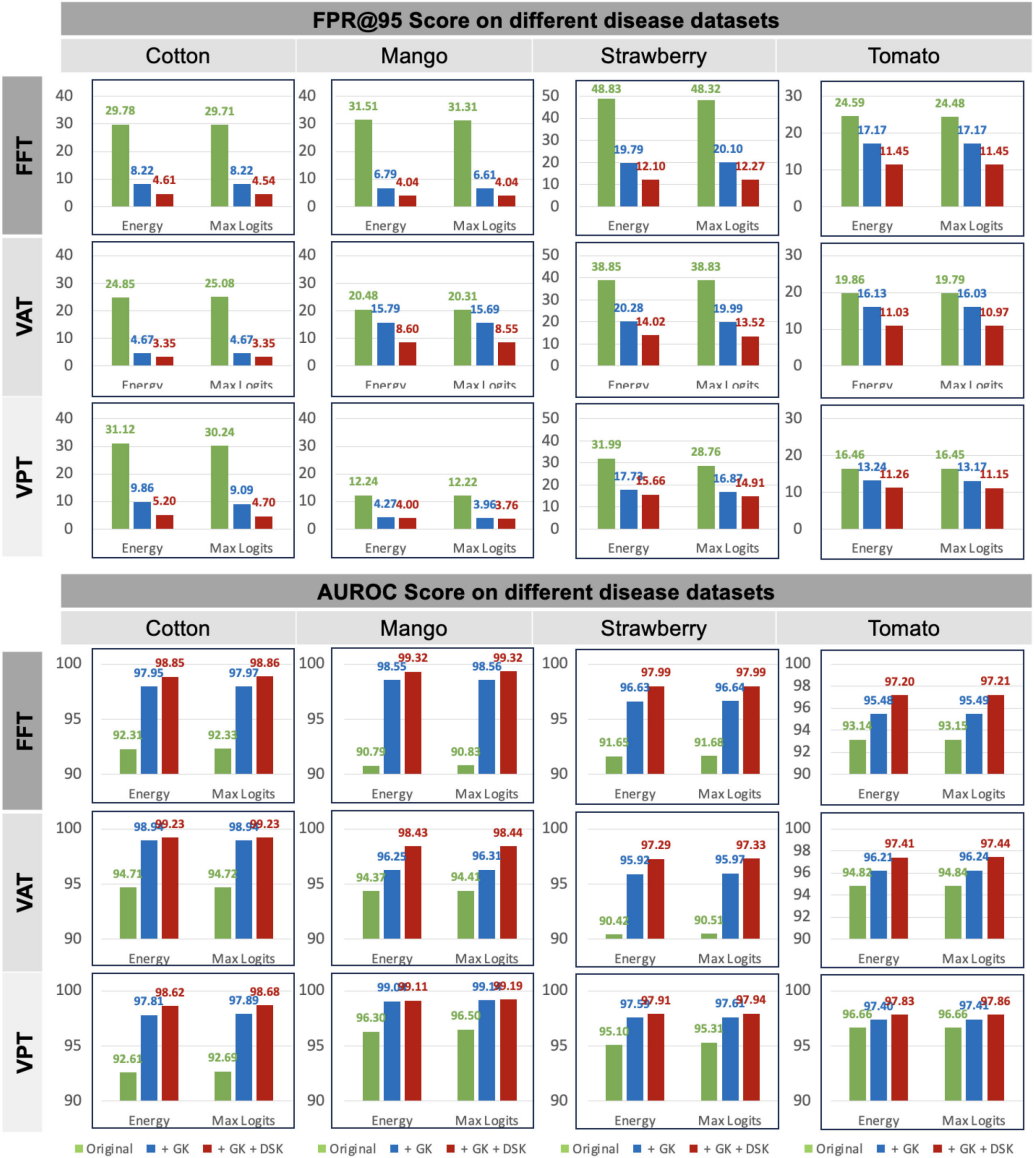
Results on four plant disease dataset in open-set settings. Dataset splits are provided in **Table 2**. The report is based on CLIP-ViT-B/16 model. Green represents baseline. The blue and red denote ensemble with different knowledge.

In the Plant Village dataset, we selected healthy leaves as the known class and treated diseased leaves as anomaly samples to evaluate the anomaly detection performance of our method. In fact, open-set tasks can also be viewed as another application of anomaly detection. In this case, some disease categories within the same plant species are considered known, while others are treated as anomalies due to the assumption that they have not yet been collected. To further validate the effectiveness of our method, we conducted experiments on four commonly used plant disease datasets: cotton, mango, strawberry, and tomato. The results are summarized in **Figure 7**. Note that the results in **Figure 7** are averaged over different experiment numbers based on various splits within the specific dataset. Please refer to **Table 2** for the corresponding experiment numbers. For example, for the Cotton dataset, we calculated the average results across three experiments.

The results demonstrate significant improvements in both FPR@TPR95 and AUROC scores when incorporating general knowledge (GK) and domain-specific knowledge (DSK). The baseline method, represented by the green bars, exhibits higher FPR@TPR95 across all datasets and frameworks, indicating a higher rate of false alarms. For instance, in the FFT framework, FPR@TPR95 for cotton starts at 29.78% but is reduced to 8.22% when combining GK and DSK. Similarly, in the VAT framework, FPR@TPR95 on strawberry decreases from 48.83% to 14.02%, and in the VPT framework, FPR@TPR95 on mango is reduced from 31.51% to 4.27%. These reductions highlight the effectiveness of our method in lowering false positive rates, particularly for challenging datasets like strawberry and tomato.

In terms of AUROC, our method achieves near-perfect scores when integrating GK and DSK. For example, in the FFT framework, AUROC on cotton improves from 92.33% to 98.85%, while in the VAT framework, AUROC on mango increases from 90.87% to 98.44%. The VPT framework demonstrates similar gains, with AUROC on tomato rising from 94.86% to 99.47%. These results underscore the robustness and adaptability of our approach across different datasets and frameworks. By explicitly integrating GK and DSK, our method enhances performance in open-set settings and ensures consistent improvements, making it highly practical for real-world anomaly detection tasks in plant disease datasets.

### 5.3 Complexity

We conduct a systematic comparison of model complexity in terms of trainable parameters, inference parameters, and inference time per image, across different architectures and fine-tuning paradigms, as shown in **Table 7**. Our method introduces a minimal number of additional trainable parameters—approximately 6K to 12K—across all training paradigms. This overhead is negligible compared to the total model size (ranging from tens to hundreds of millions of parameters), making our approach lightweight and easy to integrate. This evaluation helps quantify the trade-offs between computational cost and anomaly detection performance, especially when deploying our knowledge integration method.

**Table 7:** Comparison of parameters between baseline and our method.

Architecture		Trainable parameters	Inference parameters	Inference memory	Inference time/Image
*CNN-based* *ConvNext-base* *87.57 M*	*FFT* *FFT*(*Ours*)	87.57 M + 0.012 M 87.57 M + 0.024 M	87.57 M + 0.012 M 87.57 M * 2 + 0.024 M	3.0 G 5.9 G	1.09 ms 1.98 ms
	*VAT* *VAT*(*Ours*)	11.92 M + 0.012 M 11.92 M + 0.024 M	87.57 M + 11.92 M + 0.012 M 87.57 M * 2 + 11.92 M + 0.024 M	3.3 G 6.4 G	1.26 ms 2.15 ms
	*VPT*	0.028 M + 0.012 M	87.57 M + 0.028 M + 0.012 M	3.0 G	1.10 ms
	*VPT*(*Ours*)	0.028 M + 0.024 M	87.57 M * 2 + 0.028 M + 0.024 M	5.9 G	1.99 ms
*ViT-based vit-base-patch16-224* *85.80 M*	*FFT* *FFT*(*Ours*)	85.80 M + 0.009 M 85.80 M + 0.018 M	85.80 M + 85.80 M + 0.009 M 85.80 M * 2 + 85.80 M + 0.018 M	2.8 G 5.5 G	1.08 ms 1.98 ms
	*VAT* *VAT*(*Ours*)	0.12 M + 0.009 M 0.12 M + 0.018 M	85.80 M + 0.12 M + 0.009 M 85.80 M * 2 + 0.12 M + 0.018 M	2.8 G 5.5 G	1.11 ms 1.99 ms
	*VPT*	0.092 M + 0.009 M	85.80 M + 0.092 M + 0.009 M	3.1 G	1.10 ms
	*VPT*(*Ours*)	0.092 M + 0.018 M	85.80 M * 2 + 0.092 M + 0.018 M	6.0 G	1.99 ms
*VLM-based* *CLIP-ViT-B/16* *63.4 M + 86.2 M*	*CoOp CoOp*(*Ours*)	0.008 M + 0.006 M 0.008 M + 0.012 M	149.6 M + 0.008 M + 0.006 M 149.6 M + 86.2 M + 0.008 M + 0.012 M	5.3 G 9.1 G	2.09 ms 3.23 ms
	*CoCoOp CoCoOp*(*Ours*)	0.035 M + 0.006 M 0.035 M + 0.012 M	149.6 M + 0.035 M + 0.006 M 149.6 M + 86.2 M + 0.035 M + 0.012 M	5.6 G 9.7 G	2.37 ms 3.52 ms
	*VPT* *VPT*(*Ours*)	0.147 M + 0.006 M 0.147 M + 0.012 M	149.6 M + 0.147 M + 0.006 M 149.6 M + 86.2 M + 0.147 M + 0.012 M	5.4 G 9.3 G	2.26 ms 3.42 ms
	*VLPT*	0.156 M + 0.006 M	149.6 M + 0.156 M + 0.006 M	5.6 G	2.41 ms
	*VLPT*(*Ours*)	0.156 M + 0.012 M	149.6 M + 86.2 M + 0.156 M + 0.012 M	9.8 G	3.56 ms

We didn’t introduce any extra trainable parameters in our methods. The report is based on the Plant Village dataset with a batch size of 128.

In terms of inference, our method approximately doubles the number of parameters involved in score computation, leading to a 0.5x to 1x increase in inference-time parameters. Consequently, the per-image inference latency increases by about 1–2 ms. This time-increase remains acceptable for real-time or near real-time applications, especially considering the substantial performance gains demonstrated in previous sections. However, the total inference time remains within 1–4 ms per image, which is sufficiently fast for most practical and industrial applications, including large-scale plant disease monitoring systems.

Overall, across all training frameworks, our method introduces only minimal additional trainable parameters (0.006*M* −0.012*M*). The inference cost primarily stems from computing two independent logits and integrating them in score space, not from modifying the backbone itself. This design ensures that the proposed knowledge integration method is model-agnostic and readily deployable across various architectures without extensive retraining. Importantly, these moderate complexity increases yield significant improvements in anomaly detection performance, justifying the trade-off for real-world applications.

### 5.4 Limitations and future direction

#### 5.4.1 Limitations

While our study proposes a standardized benchmark and a practical knowledge integration strategy for anomaly detection in plant disease recognition, it has several limitations.

First, although our method is architecture-agnostic and demonstrates consistent improvements across CNNs, ViTs, and VLMs, it does not introduce a novel backbone or feature extractor. Our goal is not to outperform highly specialized state-of-the-art (SOTA) models designed for fine-grained classification [e.g., DetailCLIP Monsefi et al. (2024); Mendoza-Bernal et al. (2024)], but rather to offer a general and lightweight enhancement strategy that can be readily applied across model families. We acknowledge that integrating our approach into more complex backbones may yield higher accuracy, but this lies beyond the current scope and would require considerable computational resources.

Second, although our evaluation includes datasets collected under real-world agricultural conditions—with varying illumination, complex backgrounds, and image quality—we do not explicitly analyze how each visual factor impacts detection performance. A detailed investigation into the role of environmental variability is an important avenue for future work.

Third, our approach requires computing logits from both the pre-trained and fine-tuned branches, which nearly doubles the inference-time parameters and memory footprint. We acknowledge that this could pose challenges in edge-computing environments such as drones or mobile devices. Future research could explore model compression techniques, such as knowledge distillation or pruning, to reduce resource demands and enable more efficient deployment.

#### 5.4.2 Future direction

Despite these limitations, we believe our work provides a valuable and extensible foundation for advancing anomaly detection under few-shot and open-set conditions in plant disease recognition. A promising direction for future research is to move beyond anomaly rejection and toward a more unified open-world learning framework. Specifically, we aim to integrate open-set recognition, novel class discovery, and incremental learning into a single system. Such a system would be capable of (1) detecting unknown instances, (2) discovering and grouping new categories, and (3) continuously updating the model to recognize them. In our recent work Dong et al. (2025), we explored this direction by using prototype guided representation to discover novel plant species and diseases. We envision that combining this approach with our current method could lead to robust, real-time plant health monitoring systems that adapt to continuously evolving agricultural environments.

## 6 Conclusion

Identifying and rejecting anomalous disease categories is critical for ensuring the reliability of plant disease recognition systems. This study systematically explored the performance of different training frameworks, including CNN, ViT, and VLM, in the context of plant disease anomaly detection. Among these, the naive concept matching approach in VLM showed the poorest performance, especially when compared to using max-logits in CNN and ViT frameworks. Additionally, we examined the impact of various fine-tuning strategies on model performance and established a benchmark using the Plant Village dataset. To address the limitations of baseline methods, we proposed a general knowledge integration method to enhance anomaly detection performance. Experimental results demonstrated that our approach consistently improved performance across different training frameworks, fine-tuning strategies, sample scales, and baseline methods. Importantly, our findings highlight the critical role of selecting appropriate fine-tuning methods and pre-trained models, which can directly enhance anomaly detection without requiring additional computational resources.

In summary, this study opens a new pathway for addressing the challenge of anomaly detection in plant diseases. Our work provides a foundation for future research in developing robust and efficient plant disease detection and classification systems. We encourage researchers to further explore these methods, contributing to the advancement of reliable and scalable solutions in agricultural technology.

## Data Availability

The original contributions presented in the study are included in the article/supplementary material. Further inquiries can be directed to the corresponding authors.
